# Treatment of Pulmonary Consumption

**Published:** 1830

**Authors:** 


24. *Treatment of Pulmonary Consumption.*—In our last number we gave some account of a paper on this discourse, by the judicious and practical, Dr. Parrish of Philadelphia. In the January Number of the N. A. Medical and Surgical Journal, one of our most excellent periodicals, the Doctor has inserted another paper, from which we propose to make such extracts as will present the results of his ample experience.

My object in the present communication is to point out the course of practice, so far as this can be done in general terms, which, within my own experience, has proved most effectual as auxiliary to that great remedy, without which all the rest are nearly useless—exercise in the open air.

Though neither bleeding, nor any other mode of depletion, is at all adequate to the cure of the peculiar tuberculous disease of the lungs, which constitutes genuine phthisis, yet affections of an inflammatory nature occasionally arise in the course of the complaint, which demand an antiphlogistic treatment; as, if not relieved, they might produce either immediately fatal effects, or at least aggravate the original pulmonary disorder, and accelerate its progress.—Thus a patient with consumption may be seized with ordinary catarrh, or pneumonia, to which the previously diseased condition of his lungs predisposes him, and to which the same condition imparts a character of peculiar danger. Under such circumstances, I do not hesitate to confine my patients, to put them on a low diet, to bleed them generally and topically, to apply blisters to their breasts; in short, to put into operation the whole system of treatment adapted to cases not complicated with phthisis; always, however, bearing in mind the importance of surmounting the new danger with as little loss of strength as possible, and thus preventing the old enemy from gaining any advantage. As soon as the catarrhal and inflammatory symptoms have disappeared, I advise the patient immediately to recur to the plan of exercise. Sometimes, when there is considerable pain in the chest, with but little sympathetic affection of the constitution, it may be proper to try the effect of general or topical bleeding, without confining the patient to the house, or in any way interrupting the course of exercise which may have been prescribed: but in these cases great care should be taken not to push the lancet further than is found to accord with the general strength.

Another condition of things frequently occurs in pulmonary consumption, which requires a temporary suspension of the regular plan of treatment; I allude to hæmoptysis. A few days of rest, however, with the use of bleeding, and other suitable remedies, will be found sufficient, in the majority of instances, to remove the complaint, and to place the patient in a condition to resume his former habits. There is danger in persisting too long in the treatment for hæmoptysis; and I have myself had reason to regret the fears which have sometimes caused me to confine the invalid beyond the necessary period. With my present convictions, I would rather incur the risk of a return of the hemorrhage by too early a resort to exercise out of doors; than by long confinement and a rigid diet, break down the energies of the system, and thus remove the only barrier which opposes the advance of the tuberculous disease.

Some years ago I was called to a very delicate female, who had been attacked very suddenly with hæmoptysis, and within a very short time had discharged as much as a pint of blood from her lungs. By bleeding, rest, an appropriate regimen, and other means usually employed in such cases, I succeeded, in the course of a few days, in arresting the hemorrhage: but hectic fever now made its appearance, attended with cough, and other smptoms strongly indicative of phthisis, which, in her case, was the more to be apprehended, as she had lost her mother with that disease. The winter was approaching, and if any advantage was to be obtained from exercise, the time for commencing it could not be delayed. I laid my views of the case fairly before the family, and suggested that the choice rested with them to carry these views immediately into effect, or if they should appear too hazardous, to have the assistance of further medical advice in consultation. They were content to rely upon my judgment. The patient was recommended, notwithstanding the recent hemorrhage from her lungs, and the lateness of the season, to take exercise freely in the open air. She followed my advice, riding out in a carriage, and walking frequently, and not allowing even the cold weather to confine her to the house. The consequence was that her consumptive symptoms gradually disappeared, her strength increased, and she was ultimately restored to health: nor has she subsequently had any return of her complaint.

I have repeatedly witnessed cases of a similar character with the above, though perhaps not so striking: and it is now my uniform practice in all instances of hæmoptysis, where any tendency to consumption is displayed in the symptoms, or is apprehended from the constitution or hereditary predisposition of the patient, to recommend him very soon after the suppression of the hemorrhage to resume his outdoor avocations, and use exercise freely: nor, during many years that I have pursued this plan, have I had any reason to regret the adoption of it.

Except under the circumstances above mentioned, I am not in the habit of restricting the patient very closely in his diet. Nutritious and easily digested food is borne much better in this disease than we might imagine. The feelings of the patient himself are, in general, the best guide as to the quality and quantity of aliment which are suitable to his case. Should the use of animal food render him uncomfortable, increasing the irritation of his heart and arteries, exasperating his cough, and producing pain or uneasiness in his chest, he will readily concur with his physician in the propriety of abstaining from it; but when, instead of experiencing these effects, he finds himself refreshed and invigorated by a meal of such food, without any increase of local uneasiness, cough, or fever, his inclination should not be opposed, unless under very peculiar circumstances.—I have sometimes been much struck with the manner in which patients with consumption bear strong food. A medical gentleman who laboured under this complaint, and ultimately died of it, used to assure me, after he had abandoned all hope of recovery, that one of the greatest of his earthly enjoyments was to sit down and take a meal of that kind of plain, substantial food to which he had been accustomed when in health. He declared that he usually felt better after such a meal.

It is a well established fact, that a meagre diet, especially when combined with want of exercise, is calculated not only to bring the scrofulous predisposition into action, but even to generate the predisposition itself in a healthy and vigorous constitution. As genuine phthisis is an affection, if not identical with scrofula, at least closely analogous to it, similar results may be expected from the same cause: and it is certainly unphilosophical to attempt the cure of a disease by means which are calculated to favour its production. I never, therefore, recommend a low diet with the view of arresting or removing the tuberculous affection: it is only when hæmoptysis, or symptoms of genuine inflammation supervene, that I resort to this, together with the other antiphlogistic measures for their removal.—When this end is attained, and the proper consumptive disease only remains, I again permit the patient to consult his own inclinations as to diet, or at least allow such food as I should esteem proper for a healthy man.

On the subject of medicines in this malady, Dr. P. holds the following language:

I do not expect much from medicines in consumption. Given with the view of subverting the disease, they are manifestly injurious, as, without operating specifically upon the morbid condition of the lungs, they often produce irritation and increase debility, and thus hasten the fatal issue. But as it is desirable to preserve all the functions in as healthy a condition as possible, any accidental derangement of them should be encountered by remedies which are adapted to the removal of similar derangements under other circumstances. Thus costiveness, diarrhœa, unhealthy biliary secretion, and disordered conditions of the stomach or other viscera, should be treated with appropriate medicines, with this reference only to the chief disease, that it is likely to be aggravated by the continuance of these morbid affections, and that, in the means employed for their removal, its influence in repressing the vital energies, and exhausting the system should not be overlooked. Occasion for the use of medicine is also afforded by the distressing symptoms which attend the progress of the malady, and which a regard for the comfort of the patient requires that we should try to palliate, as we are unable altogether to remove them. Opiates, and other anodynes, to relieve cough and allay irritation, with various pectoral medicines to assist or promote expectoration, are occasionally useful. Sometimes gentle tonics are advantageous in counteracting the debilitating and wearing influence of the disorder, especially in its more advanced stages. Of these I have found none to answer so well as the infusion of the wild cherry-tree bark. It promotes digestion, and gives tone to the system generally, without producing that irritation of stomach, and constriction of the breast, which are apt to result from the use of Peruvian bark in the same condition of things. The simple bitters, such as quassia, gentian, and columbo, may also be employed: and when a powerful tonic is required, the sulphate of quina will be found to answer a better purpose than the bark in substance. I occasionally prescribe Fowler's solution when the chills are very distressing; but always with caution, and never for a longer period than two or three days at a time. A small blister kept open for some time, and renewed on different parts of the chest successively, is sometimes advantageous by giving an external direction to the disease.

Decidedly as we concur with Dr. P. in these views, when true tubercular Ph thesis is in our contemplation, we must be permitted to express a doubt, whether they are correct in reference to the other varieties of consumption; such as chronic bronchitis, chronic pneumony and vomica. In these affections, there is no specific morbid action, and hence they are (in their curable stages) to be treated by ordinary antiphlogistic and sedative means.

